# Between host country and homeland: a grounded theory study on place of dying and death in migrant cancer patients

**DOI:** 10.1186/s12904-025-01821-3

**Published:** 2025-08-08

**Authors:** Simona Sacchi, Loredana Buonaccorso, Silvia Tanzi, Giulietta Luul Balestra, Luca Ghirotto

**Affiliations:** 1 Palliative Care Unit, Azienda USL-IRCCS di Reggio Emilia, Reggio Emilia, Italy; 2 Psycho-Oncology Unit, Azienda USL-IRCCS di Reggio Emilia, Reggio Emilia, Italy; 3 Qualitative Research Unit, Azienda USL-IRCCS di Reggio Emilia, Reggio Emilia, Italy

**Keywords:** Cancer, Migrant patients, Decision-making, Place of death, End-of-Life, Palliative care, Advance care planning, Transnationalism, Grounded theory

## Abstract

**Background:**

Migrant cancer patients face unique challenges in end-of-life decision-making. One key yet underexplored aspect is the decision-making process surrounding the place of dying and death. This study explores the factors influencing these decisions involving migrant cancer patients in Italy.

**Methods:**

A constructivist grounded theory approach was employed. Data were collected through semi-structured interviews with 28 participants (patients, family members, cultural mediators, and other key informants, some from a previous grounded theory study). Theoretical sampling guided participant selection for this study, and data analysis followed an iterative coding process, leading to the development of a conceptual model.

**Results:**

The decision-making process was conceptualized as a gradient field in which three forces interact to shape outcomes: (1) Healthcare as an Attracting/Holding Force—quality medical care in Italy encouraged patients to stay, though its influence diminished as curative treatments ended; (2) Bureaucratic and Financial Barriers as an Obstructing Force—challenges related to residency, access to care, and financial constraints often complicated decision-making, particularly for those considering repatriation; and (3) Social Networks as a Stabilizing Force—the strength of familial and community ties in host country and homeland played a decisive role in shaping preferences. A fundamental opacity about terminality was present, with limited communication and awareness regarding prognosis, further expanding the circle of decision-makers.

**Conclusions:**

Palliative care professionals should recognize the importance of transnational networks, consider bureaucratic barriers, and encourage open discussions regarding place of dying and death decision. To navigate these complexities, we propose a question guide for specialists during consultations. This tool aims to enhance culturally sensitive communication, promote shared decision-making, and address the challenges of cross-border end-of-life care. By accommodating transnational ties, palliative care services can better align with the realities of migrant cancer patients, fostering equitable and dignified end-of-life care.

**Supplementary Information:**

The online version contains supplementary material available at 10.1186/s12904-025-01821-3.

## Background

In recent decades, global migration has steadily increased, shaping demographic and healthcare landscapes in destination countries. Europe has seen considerable migratory shifts, with 44.7 million individuals born outside the European Union (EU) living in EU countries, accounting for 9.9% of the total EU population (Jan 1, 2024) [1, 2]. The drivers of migration to Italy are multifaceted, including geopolitical instability, economic opportunities, and climate change [3, 4]. As of January 2023, over 5 million foreign citizens resided in Italy, primarily from other European countries (EU and non-EU, 50%), North and West Africa (22.2%), and Asia (22.6%) [5]. Emilia-Romagna has the highest incidence of foreign citizens, with nearly 13 per 100 inhabitants.

Migrants arriving in Italy present diverse health needs, necessitating that healthcare systems adapt and ensure equitable access to services. In the context of cancer, while newly arrived migrants may have lower cancer incidence rates, over time, they may experience an increased risk due to lifestyle-related risk factors, delayed screenings, and limited healthcare access, necessitating tailored healthcare interventions [6–9]. Addressing the evolving epidemiological trend calls upon the preparedness of palliative care (PC), given its critical role in supporting migrant populations in situations of significant vulnerability.

PC, as defined by the 2020 international consensus [10], is the active, holistic care of individuals of all ages experiencing profound health-related suffering due to severe illness, particularly those near the end of life (EoL). PC aims to improve patients’ and their families’ quality of life (QoL) by addressing physical, emotional, social, and spiritual needs [11].

The relationship between PC provision and ethnic minorities presents significant issues and disparities that merit consideration both globally and within Europe [12, 13]. Migrant patients in PC settings represent a complex group, not only due to their clinical needs but also because of the interplay of social, economic, cultural, and spiritual factors that influence care provision [14].

Existing research highlights inequities in PC access, suboptimal symptom management, and disparities in pain control when compared with native populations [13, 15–17]. These disparities become even more pronounced at the EoL, necessitating urgent efforts to improve inclusivity in PC services [18, 19]. Language barriers and health literacy may reduce patients’ ability to understand their diagnosis, prognosis, and available care options [20]. Additionally, professionals may lack training in cultural competence, leading to inadequate recognition of the cultural factors shaping EoL decision-making [21]. Research from our group previously highlighted that while healthcare professionals (HPs) in Italy are trained in EoL care, they often lack formal education on intercultural communication and culturally adapted care strategies [22].

The complexities of managing EoL care for migrants can lead to difficulties addressing needs specific to this population. A crucial element of these needs is where care and death occur [23], and whether it aligns with patients’ preferences and desires. The concept of congruence, or the alignment between a patient’s preferred and actual place of death, is a key indicator of PC quality [24]. A systematic review identified illness-related factors, individual preferences, and environmental conditions as key predictors of congruence, with pain control, caregiver availability, and discussions with HPs being particularly influential [25].

In the existing literature, “place of death” often refers to clinical settings such as a hospital, home, or hospice [25–29]. In this context, research shows that ethnic minorities are more likely to die in hospitals than in-home or hospice settings, often due to limited knowledge about PC services or inadequate information about how to access them [30, 31]. However, for migrant patients, this notion extends beyond institutional categories and takes on a broader, more existential dimension [23]. In our study, “place” is conceptualized at a macro level, referring not only to the site of care but also to the country in which dying and burial will occur. This macro-level understanding reflects how the place of death is deeply entangled with questions of sense of belonging, identity, and transnational attachments. Migrants may possess a unique sense of location and dislocation [23, 32], shaped by the lived experience of movement, the restructuring of familial and social networks across borders, and the emotional significance of homeland and host country [33, 34]. This transnational sense of “home” influences where they hope to receive EoL care and, ultimately, where they prefer to die and be buried. Recognizing the place of death as both a geographical and symbolic site is essential for PC professionals seeking to provide culturally congruent and meaningful care. Yet, this perspective remains underexplored in PC literature and practice, despite its significance for many patients with a migration background [23].

In addition to medical and logistical concerns, cultural beliefs play a role in shaping EoL care and possibilities. Migrant patients may experience a “double home experience” [35], feeling torn between their homeland and their new country, which complicates processing about their preferred place of care, death, and burial [36]. The “double home experience,” as described in the literature on migrant EoL care, refers to the ambivalent attachment to both the host country and the homeland that many migrants experience when facing terminal illness. This notion aligns closely with Abdelmalek Sayad’s sociological concept of the double absence [37], which frames the migrant as simultaneously absent from the society of origin and marginal within the society of arrival. In the context of terminal illness, this dual dislocation becomes particularly salient: migrants may feel they do not fully belong to the healthcare, cultural, or social systems of the host country, yet al.so experience estrangement or logistical inaccessibility in returning to their country of origin to die. The decision-making process surrounding the place of death thus becomes more than a logistical or clinical matter—it is a deeply existential negotiation of belonging, identity, and relational continuity across borders [38].

In other words, the interplay of home-related sense, identity, and family profoundly shapes how migrant populations experience institutional systems of care and death. These dimensions are not merely individual concerns but influence—and are influenced by—structural frameworks and professional practices within healthcare systems. The experience of serious illness can intensify ambivalence about where “home” truly is, particularly when individuals fear dying far from loved ones or being unable to participate in culturally meaningful funeral rites [39]. Such fears are often compounded by a heightened awareness of displacement and impermanence, particularly among those with transnational ties. However, institutional systems are not always equipped, and HPs may struggle to accommodate diverse cosmologies of dying and belonging, especially when standardized protocols leave little room for adaptation [38, 40].

This study underscores the necessity of incorporating empirical evidence into the discourse surrounding the preferred place of dying and death for migrant cancer patients (MCPs), especially in Italy, where this issue has yet to be rigorously explored.

Italy has a universal public healthcare system (Servizio Sanitario Nazionale, SSN), primarily funded through general taxation. Emergency care is available to everyone, regardless of legal status. In oncology and PC, services are typically provided through public hospitals, hospice care facilities, and community-based networks, including home-based PC units. However, access for MCPs can be uneven due to bureaucratic barriers, lack of residence permits, language difficulties, and unfamiliarity with the healthcare system [41]. These factors can delay entry into care pathways, limit continuity, and complicate EoL decision-making, particularly when navigating between countries or planning for repatriation [41].

This study aims to equip PC specialists with updated insights for enhancing the overall quality of care delivered to this population [14]. This study explores, in particular, the factors influencing the decision-making process regarding the place of care and death for MCPs in Italy. These include whether to remain in Italy, return to the country of origin, or move between the two, as well as preferences regarding repatriation of the body.

The research question guiding the process was: What factors influence the decision-making process regarding the place of dying and death for MCPs?

## Method

### Study design

This study adopted a constructivist grounded theory (GT) approach [42] to exploring decision-making processes in patients with migrant backgrounds in their last phase of life, assisted by the Palliative Care Unit (PCU) at Azienda USL-IRCCS of Reggio Emilia.

Constructivism in qualitative research is an epistemological perspective that views knowledge as co-constructed through the interactions between researchers and participants, emphasizing the subjective meanings individuals assign to their experiences [43]. As an epistemological lens, constructivism posits that reality is not objectively discovered but subjectively constructed through social interactions. In this study, our theoretical lens was also informed by a commitment to social justice, as emphasized by Charmaz [44, 45], who argued that constructivist grounded theory can serve as a vehicle for amplifying marginalized voices and addressing structural inequities. This orientation guided our reflexive engagement with participants and our aim to illuminate the complex, often overlooked experiences of migrant patients at the end of life.

In particular, Charmaz’s methodology guided our iterative data collection and analysis process, allowing for flexibility and responsiveness to emerging insights. The researchers’ positionalities were acknowledged as integral to the research process, and reflexivity was maintained throughout to ensure transparency and rigor. This reflexive approach ensures that the research process remains dynamic and adaptable, recognizing that the data and the resulting theory are shaped by the interpretations and meanings shared during the research process [42].

The research builds upon data previously collected for a constructivist GT study on care processes for MCPs at the EoL [22]. The previous constructivist grounded theory study titled “Achieve the best while rushing against time” explored the perspectives of healthcare professionals, family members, and stakeholders on the care of MCPs at the EoL. That study identified key challenges in intercultural communication, cultural sensitivity, and systemic navigation, culminating in a model emphasizing the urgency and emotional labor involved in providing care. During re-analysis of the original dataset, the theme of decision-making regarding the place of dying and death was a recurrent but underdeveloped concern. This prompted formulating a new research question and extending the original study through additional theoretical sampling and interviews. The current study thus represents a deepening of the original inquiry, focusing specifically on the transnational, bureaucratic, and social dynamics that shape EoL decisions for MCPs. The previous study did not involve any patients, indicating the potential for the GT’s findings to be expanded in future research. Consistent with the adaptability inherent to GT methodology [42, 46], we extended data collection by incorporating new participants (patients and other key informants) and re-analyzing earlier data to deepen theoretical insights to answer the new research question.

A distinctive feature of GT is its modifiability, which refers to the method’s inherent flexibility to evolve as new insights and understandings develop [47–49]. This adaptability is a key strength of the approach, as it allows researchers to revisit and refine previously analyzed data, incorporate new participants, and expand the theoretical framework. Modifiability ensures that the theory remains responsive to the complexity and variability of the studied social phenomena, providing a deeper and more nuanced understanding. For example, in this study, the flexibility of GT enabled the re-analysis of earlier data and integration of additional participants to develop further and refine the theoretical model. This approach aligns with the iterative nature of GT, where theory is built through a continuous process of data collection, analysis, and refinement. This ensures that the emerging theory remains grounded in the realities of participants’ experiences.

To report the study, we adhered to the recommended Standards for Reporting Qualitative Research (SRQR) [50] (see supplementary material).

### Setting

The research setting was the PCU of the Azienda USL-IRCCS of Reggio Emilia, Italy. The PCU is located within the Comprehensive Cancer Center, in a general hospital in Reggio Emilia, Italy, serving a provincial catchment area of over 530,000 inhabitants. The Cancer Hospital has 200 beds and offers diagnostic, therapeutic, rehabilitation, supportive, and PC for cancer patients.

The PCU is a specialized, hospital-based consultative service without dedicated inpatient beds. The PCU staff assists outpatients and inpatients with advanced oncological or chronic progressive diseases, including neurological and respiratory diseases. Its mission is to perform clinical, training, and research activities in PC. Established in 2013, the unit is staffed by three senior physicians and three advanced practice nurses. Additionally, five psychologists from the hospital’s Psycho-Oncology Unit collaborate with the PCU, providing clinical consultations, facilitating staff training, and participating in research and educational initiatives in the PC field. In 2024, the PCU conducted 2650 consultations, including 647 initial consultations and 219 family conferences. The percentage of cancer patients assessed by the PCU out of the total number of cancer patients hospitalized yearly was 14% [51].

Regarding MCPs, data from the PCU in 2022 indicated that 43 of the 534 patients (8%) had a migration history, mainly due to work-related or family reunification. These individuals originated from countries facing social vulnerabilities, which included limited access to services in their home country, language barriers, and socioeconomic disparities in their host country. This percentage remained the same in 2023, with 45 of 541 patients (8%). In 2024, the PCU assisted 54 MCPs (inpatients and outpatients), representing 9%.

### Sampling and recruitment

Sampling and data collection were conducted simultaneously, a hallmark of GT methodology. Following a re-analysis of data from the initial GT study [22], participants for this study were selected through theoretical sampling, which involves identifying individuals whose experiences help refine emerging categories and enhance the theoretical framework [42]. In this study, initial sampling referred to the participant group from our previous GT study [22], which explored the broader care processes for MCPs. This dataset, comprising 12 interviews with 14 participants, served as the foundation for the current inquiry. When a new research question emerged—specifically about decision-making related to the place of death—we used theoretical sampling to recruit more participants capable of refining and deepening the categories based on this updated research goal. The unit of analysis for this second study focused on MCPs at the EoL, along with their family members, other close proxies, and key informants involved in decisions regarding the place of death and burial. Inclusion criteria for patients were:

- Advanced metastatic cancer with a clinical prognosis of weeks to months.
- Age ≥ 18 years.
- Migrant background from countries commonly associated with structural and social vulnerability in the Italian healthcare context. This included individuals from regions where migrants often face barriers such as limited access to services, language and cultural differences, and socioeconomic disadvantage (this classification was not based on formal World Bank income categories but rather on the practical realities of marginalization experienced by these populations within the host country).
- Enrolled in the PCU.
- Ability to communicate in Italian or willingness to involve a cultural mediator during data collection.
- Signed informed consent.

All participants were recruited through the PCU specialists, who include a clinical psychologist as part of the multidisciplinary team. Patients involved in the study were already under the care of this psychologist, who routinely provides emotional support, particularly following the communication of bad news or during discussions involving prognosis and EoL issues.

Other participants were included if they:

- Were ≥ 18 years old.
- Developed a meaningful relationship with the patients or their networks, or had information about the decision-making process around the place of death. Particular categories were:
  - Funeral operators since they have defined as important players, as noted in previous interviews with family members and cultural mediators. While they are usually not seen as part of the healthcare team, family members, healthcare professionals, and cultural mediators identified them as crucial figures who often engage before death, especially when repatriation is involved.
  - Cultural mediators. In the Italian healthcare system, cultural mediators are professionals who go beyond linguistic translation to facilitate mutual understanding between healthcare providers and patients from diverse cultural backgrounds. Unlike standard interpreters, cultural mediators are trained to interpret both language and cultural context, helping to navigate differences in health beliefs, communication styles, and expectations. Their role is essential in supporting equitable, culturally sensitive care [52], particularly in EoL settings.
- Could communicate in Italian or agree to the presence of a cultural mediator.
- Signed informed consent.

If patients were deceased, family members were recruited within 2–4 months post-mortem.

Potential participants were approached as follows:

- As for patients, they were introduced to the study during in-person meetings with PCU professionals. If necessary, a cultural mediator assessed linguistic competencies.
- The principal investigator contacted the other participants via phone or in-person meetings (S.S.). The need for cultural mediation was similarly evaluated.

With participants’ consent, researchers scheduled interviews at times and places convenient for them. Participants were not compensated financially for their involvement. Interview guides in participants’ native languages were available upon request.

The final sample and related characteristics are shown in Table 1.

**Table 1:** Participants’ characteristics

Code	Gender	Age (years range)	Type or Role	Sampling	Background	Notes
P01	W	61–70	Widow	Initial	Chilean	Immigrated with the husband for work. Permanent resident status.
P02	W	41–50	Widow	Initial	Moroccan	Immigrated with the husband for work. Non-permanent resident status.
P03	W	31–40	Daughter	Initial	Moroccan	Born in Italy. Italian citizen.
P04	W	31–40	Sister-in-law	Initial	Albanian	Immigrated for work. Non-permanent resident status.
P05	M	41–50	Funeral operator	Initial	Italian	
P06	M	41–50	Funeral operator	Initial	Italian	
P07	W	41–50	Psychologist	Initial	Italian	She was working at the funeral agency, providing psychological support to employees and clients
P08	W	31–40	Social worker	Initial	Italian	
P09	W	51–60	Cultural mediator	Initial	Albanian	
P10	W	51–60	Cultural mediator	Initial	Russian	She served as cultural mediator for Russian and Ukrainian patients
P11	W	41–50	Cultural mediator	Initial	Moroccan	
P12	M	61–70	Charity director	Initial	Italian	
P13	W	41–50	Association member	Initial	Albanian	
P14	W	31–40	Social worker	Initial	Italian	
P15	W	61–70	Cultural mediator	Theoretical	Chinese	
P16	M	61–70	Patient	Theoretical	Albanian	Immigrated for work almost 30 years before, diagnosed with thyroid cancer. He applied for Italian citizenship.
P17	W	51–60	Patient	Theoretical	Georgian	Immigrated for work almost 20 years before, diagnosed with pancreatic cancer. Non-permanent resident status.
P18	W	61–70	Patient	Theoretical	Moroccan	Immigrated to reunite with family about 40 years ago, diagnosed with pancreatic cancer. Non-permanent resident status.
P19	M	61–70	Patient	Theoretical	Albanian	Immigrated for healthcare for several months, diagnosed with lung cancer. Non-permanent resident status. A cultural mediator was present during the interview.
P20	M	51–60	Patient	Theoretical	Moroccan	Immigrated for work almost 30 years before, diagnosed with lung cancer. Permanent resident status. A cultural mediator was present during the interview
P21	M	51–60	Patient	Theoretical	Ghanian	Immigrated for work almost 20 years before. Non-permanent resident status. He returned to Ghana where he found out the cancer. He came back to Italy for healthcare, as he was diagnosed with gastric cancer. A cultural mediator was present during the interview
P22	W	61–70	Patient	Theoretical	Georgian	Immigrated for work almost 20 years before, diagnosed with biliary tract cancer. Non-permanent resident status.
P23	M	31–40	Nephew	Theoretical	Moroccan	Born in Italy. Permanent resident status.
P24	W	21–30	Widow	Theoretical	Albanian	Immigrated for healthcare in the last couple of years. Non-permanent resident status.
P25	W	31–40	Granddaughter	Theoretical	Moroccan	Born in Italy. Italian citizen.
P26	W	51–60	Funeral operator	Theoretical	Italian	
P27	M	51–60	Funeral operator	Theoretical	Italian	
P28	M	41–50	Funeral operator	Theoretical	Italian	

### Data collection

We expanded on the previous GT by conducting semi-structured interviews from March 2023 to November 2024. We followed a flexible structure typical of the intensive interview style [42, 53], which is characterized by open-ended, flexible, and conversational questioning that allows participants to share detailed narratives while enabling the interviewer to explore emerging themes through probing. This approach aligns with Kvale’s view of qualitative interviews as meaning-making conversations [53], where knowledge is co-constructed through interaction between interviewer and participant. The interview guide was developed in line with the principles of GT, without a pre-assumed theoretical framework. The topics and corresponding exemplifying questions were as follows:

1. Accounts of the Migration Experience (Can you share your migration story? Can you tell us about your experiences here in Italy? ).
2. Current Experience (How are you experiencing this moment? How are you spending your days? ).
3. Priorities (What is important to you right now? ).
4. Future Organization and Decisions (What do you plan to do shortly? Do you have any practical wishes regarding your future? ).

To ensure ethical sensitivity, we began with open-ended life-narrative questions, allowing participants to share what they felt was most important. This approach respected their emotional state while enabling themes related to EoL care and place of death to emerge organically.

The same subjects were modified and discussed during interviews with the other participants, incorporating adjustments to capture their viewpoints. For them, interview prompts were adapted to explore EoL topics more directly and in alignment with the research objectives.

Two researchers (an interviewer and an observer) conducted the interviews, which were audio-recorded and transcribed verbatim. Field notes captured the emotional and relational aspects of the interactions. Three researchers (L.B., L.G., S.S.) alternated conducting the interviews.

### Data analysis

Interviews were anonymized and enriched with socio-demographic data. We followed Charmaz’s indications for coding [42], entailing three steps of increasing abstraction: initial/open coding, focused coding, and theoretical coding. The coding process was focused and systematically aligned with the principal concerns expressed by the participants, following the prescribed methodological indications [42, 48, 49]. We began with initial coding by analyzing the first set of interviews drawn from the previous GT study. Namely, initial coding applied inductive constant comparison methods to data from the first study, which involved 12 interviews with 14 participants (two interviews involved two participants). This phase aimed to identify new preliminary categories aligned with the updated research question. Two researchers (S.S. and L.B.) conducted coding independently, and then met regularly to compare interpretations, discuss emerging codes, and resolve discrepancies through consensus. Disagreements were addressed through reflective dialogue, drawing on memos and contextual notes to ensure that coding decisions remained grounded in participants’ narratives. This phase yielded 16 subcategories.

As new interviews were conducted, we proceeded with focused coding, identifying and applying the most significant and recurrent codes to synthesize broader patterns and meanings. Eight additional interviews were involved, and the categories were iteratively refined into five provisional categories (related to the experience of being assisted by HPs, sense of belonging, financial constraints, bureaucracy, and awareness of terminality). Focused coding was again conducted collaboratively, with regular peer debriefings involving a third researcher (G.L.B.) to enhance analytical rigor and ensure intercoder reliability.

Theoretical coding followed, integrating and relating the focused codes to develop a coherent and interpretive conceptual framework. At this stage, all four core researchers (S.S., L.B., G.L.B., and L.G.) participated in analytic discussions to construct and refine the emerging theoretical model. Disagreements or alternative interpretations were documented and discussed until a shared understanding was reached.

Subsequent interviews (*n* = 4, involving 6 participants) were conducted within a theoretical sampling framework to test and refine the emerging model. Theoretical saturation was considered achieved when no new conceptual insights emerged. Final validation of the categories and core category was achieved through group discussions and iterative data review, ensuring the model was grounded in the data and conceptually robust. The coding tree is outlined in the supplementary materials.

### Reflexivity, rigor, and memoing

The research team comprises an interprofessional and multidisciplinary group, bringing together diverse expertise that enriched the analytical process and facilitated a more abstract conceptualization of the data. The team includes two PC physicians (S.S. and S.T.), a psychologist and psychotherapist (L.B.), a medical anthropologist (G.L.B.), and a qualitative methodologist and medical sociologist (L.G.).

Beyond disciplinary backgrounds, we also reflected on how other aspects of our positionality—such as gender, linguistic background, and lack of direct migration experience—may have influenced the research process. Most interviews were conducted by female researchers with psychological or palliative care expertise, while a few were conducted by a male researcher with a sociological background. These dynamics may have shaped participants’ comfort levels and the types of narratives they shared, particularly in emotionally sensitive conversations. None of the authors had personal migration experience, which may have introduced limitations in interpreting culturally situated narratives. However, this was addressed through a deliberate stance of openness, active listening, and attentiveness to participants’ meanings and categories, in keeping with the principles of GT.

Furthermore, the team had long-standing collaborative relationships with the cultural mediators involved in the study, allowing for reflexive engagement with the interpretive layers added by their presence during some interviews. All researchers and interviewers, as were most participants, were native Italian speakers, though linguistic mediation was employed when needed. These intersecting factors were regularly discussed in team meetings and integrated into the analysis through memo-writing and joint reflection.

As to data analysis, this plurality of perspectives allowed for a more comprehensive interpretation of the findings, integrating clinical, psychological, anthropological, and sociological insights. Given their role in patient care, S.S. and S.T. established initial contact with participants but did not conduct patient interviews to minimize potential desirability bias. Instead, the other authors conducted the remaining interviews, ensuring a more impartial data collection process. However, S.S.’s presence in the clinical setting contributed to the successful enrollment of participants, as patients and families perceived her as a trusted figure. The research team also reflected on its positionality concerning the study topic. The research team possessed different levels of expertise in migration studies and their implications, as well as in clinical and healthcare settings. This disciplinary asymmetry required ongoing reflexivity to ensure that migrant patients’ experiences were interpreted within an appropriate socio-cultural framework, rather than exclusively through a clinical lens. The research team engaged in regular discussions to critically examine their assumptions and mitigate potential biases, ultimately fostering a more nuanced and context-sensitive understanding of the EoL experiences of MCPs. Disagreements during the analytic process were resolved collaboratively, and an audit trail was maintained to ensure transparency and rigor.

Memo-writing played a key role in deepening our interpretation of the data, supporting ongoing abstraction through shared reflections and team discussions. Memos were particularly useful in examining the role of cultural mediators, who assisted in three interviews to ensure linguistic and relational accessibility for participants not fluent in Italian. While their presence was essential to facilitate trust and communication, it also introduced an additional interpretive layer that shaped how narratives were constructed. To address this analytically, we held debriefing sessions. We wrote targeted memos after each mediated interview, reflecting on the influence of mediation on meaning-making, including how relational or cultural dynamics might have shaped certain expressions or silences. Notably, interviews involving mediators were often shorter and occasionally interrupted, which may have affected the depth of the dialogue.

We adhered to Charmaz’s GT criteria of credibility, originality, resonance, and usefulness [42] to ensure this study’s rigor. We also incorporated additional trustworthiness strategies, as Guba and Lincoln [54] outlined. Credibility was achieved through methodological triangulation, which collected rich, in-depth data from semi-structured interviews, observations, and memos. Data were transcribed verbatim and analyzed using line-by-line coding. Multiple researchers independently analyzed the data, enhancing credibility through collaborative discussions. Reflexive practices were integral throughout the research process; researchers maintained reflexive memos to document their positionality and its potential influence on data interpretation [55]. These memos were shared and discussed with an external colleague (S.T.), promoting critical dialogue and ensuring consistency in analytical rigor. This process strengthened the study’s trustworthiness and contributed to its usefulness by ensuring the emerging theoretical model accurately reflected the participants’ experiences.

### Ethical considerations

The Provincial Ethics Committee of Reggio Emilia approved the initial study (protocol No. 2021/0150631), which was amended in February 2023 (906/2021/OSS/IRCCSRE). Participants provided oral and written informed consent, including authorization for sensitive data processing. Interviews were conducted with sensitivity, and the research team was trained to recognize signs of distress and respond appropriately, including referring participants back to the psychologist if needed.

## Results

The findings are based on data collected from 28 participants. Of these, 14 participants were included from the previous GT. In this study, we initially approached 18 individuals. Still, four declined to participate: one patient who experienced a critical fall before the interview, a grieving son, and a daughter and cousin who became unreachable for contact.

For this study, we conducted 12 interviews, one of which involved 3 participants. Three interviews required linguistic support from cultural mediators, specifically for Albania, Morocco, and Ghana patients. The average duration of the interviews was 52 min, ranging from 18 to 130 min.

### Grounded theory findings

The findings are organized around four major analytic categories that we developed from the analysis of the interviews. These categories reflect the key factors shaping how decisions are made about the place of care and death for patients living far from their country of origin.

The first category, Opacity of Terminality, describes how the EoL phase is often not acknowledged or communicated to the patient. In many cases, neither patients nor their families spoke openly about terminal illness, which affected their ability to plan ahead or make informed decisions. The second category, Healthcare as an Attracting/Holding Force, focuses on the healthcare system’s role, particularly access to high-quality care in Italy, as a factor that encouraged patients to remain in the host country. This was especially true for those who had migrated specifically for medical treatment or lacked access to similar care in their country of origin. The third category, Bureaucratic and Financial Barriers as an Obstructing Force, addresses patients’ challenges in accessing benefits, managing documentation, and considering repatriation. These structural barriers played a significant role in limiting feasible options for care and post-mortem arrangements. The fourth category, Social Networks as a Stabilizing Force, captures the direct and indirect influence of family members, both in Italy and abroad, on patients’ decisions. The emotional, practical, and cultural support offered by these networks was critical in shaping preferences around staying, returning, or being buried in a specific location.

These four categories describe a complex decision-making process that unfolds in a transnational context. Based on these findings, we developed an overarching conceptual model to explain how these factors interact, often in tension. In this sense, those categories’ interplay can be conceptualized as a gradient field in which the magnitude and direction of these competing categories determine the outcome. Analogous to a spatial gradient, the varying intensity of these forces influenced the direction of movement, whether toward remaining in the host country, returning to the country of origin, or realizing the desire for more fluid, circular migration patterns that allow for periodic travel between the two locations for treatment and care. These forces, like the parameters of a gradient, created a perceived proximity or distance to the place of death, shaping the experience of terminal illness within a migratory trajectory. Categories and related sub-categories are summarized in Table 2.

**Table 2:** Categories and Sub-categories

Categories	Sub-categories
1. Opacity of terminality	Poor awareness of terminal phase of illness Attitude of protecting loved ones Failure of migration project Difficulty in communication and comprehension
2. Healthcare as an Attracting/Holding Force	Accessibility and quality of Italian healthcare Difficulty in deciding about palliative interventions Transnational circulation of care
3. Bureaucratic and Financial Barriers as an Obstructing Force	Complexity of administrative processes The role of the legal status Economic constraints Communities’ solidarity Bureaucratic obstacles to EoL options
4. Social Networks as a Stabilizing Force	Family members near and far Decision-making of family members Cultural and spiritual aspects Proximity to significant others

**Fig. 1:**
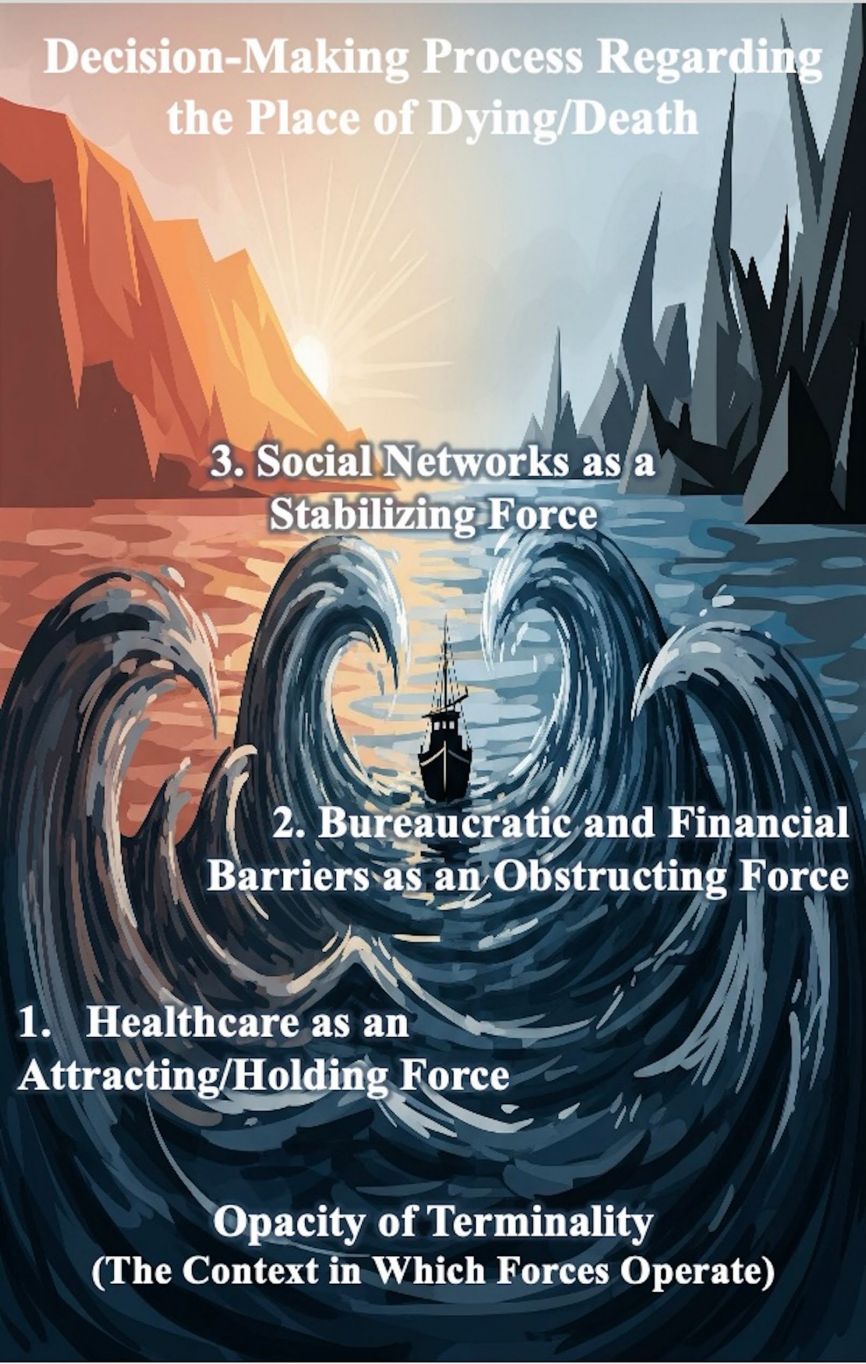
The GT model

The core category, “settling the gradient’s forces”, denotes the outcome of the decision-making process. This concept was a collective concern among all participants engaged in reconciling the forces of this decisional gradient, which refers to the point at which the competing forces influencing the circumstances surrounding the dying person attain a state of equilibrium. The visual model in Fig. 1 shows this conceptualization.

### The opacity of terminality: the context in which forces operate

A critical factor shaping the decision-making process was the lack of clarity or open discussion about the terminal stage of illness. This opacity of terminality arose from multiple factors, including the protective stance of family members, the patients’ hopes of returning to their homeland, their relationship with their migration project, and structural barriers such as delayed or absent cultural/linguistic mediation.

“We never told him exactly what was happening… we didn’t want to take away his hope that he could go back home” (P04, sister-in-law).

“In this case, the choice was difficult because he, not knowing the language, was always attended by a close relative whenever he went to the hospital. However, he was unaware of what was happening. It was a tough decision to either make him aware of everything, knowing that there was no longer any chance, or to say nothing until the end over” (P25, granddaughter).

The delay or lack of timely professional cultural and linguistic mediation or cultural brokerage exacerbated the difficulty of discussing terminality, broadening the circle of decision-makers beyond the patient and immediate family.

“There was no mediator, so all the conversations were fragmented… it became the doctors, the relatives, and eventually the whole extended family who decided” (P09, cultural mediator).

When they were present, cultural mediators usually respected families’ preference for not sharing information.

“We often find ourselves in a tough situation when it comes to sharing difficult news with our elderly parents, especially when we know that their condition is quite serious and time may be limited. The offspring is aware but chooses to keep the truth from them. In one recent situation, the whole family made a trip back to China, as the daughter understood her parents’ condition was critical and felt it was important to have them close during this time” (P15, cultural mediator).

When clarity about terminal illness replaced uncertainty, decision-making became easier. One social worker articulated this well:

“We relied on professional mediators to ensure she (*the patient*) understood her condition to plan her return home” (P14, social worker).

Many migrant patients’ original motivation for migration was to improve their QoL and provide economic support to their families in their country of origin. This life trajectory often made accepting terminality particularly difficult, as it signified a rupture with the fundamental nature of the migration project itself.

“He came here for work, not to die. He always said: ‘I will go back when I am better’” (P15, cultural mediator).

This opacity in acknowledging terminality influenced how other forces, such as healthcare, social networks, and bureaucracy, were perceived and traversed.

### Healthcare as an attracting/holding force

The accessibility and quality of healthcare in Italy, in general, and in the research context, acted as a positive decisional gradient, exerting a strong pull toward coming and staying. Some individuals relocated to Italy explicitly to receive treatment, often leveraging pre-existing family networks:

“I came here because my brother was already living in Italy, and I trusted the Italian healthcare… I knew I could get better care [for her husband]” (P24, widow).

“I knew I wouldn’t be able to afford care in my country, so I came to Italy, at least for now” (P19, patient).

Patients or their families often chose to remain in Italy as long as curative treatments were perceived to be available, highlighting the role of ongoing medical interventions in sustaining their stay:

“The doctors assured us that there was no possibility of a cure, so we decided to take him to Morocco as long as he could… as long as he could stand, and then he died there” (P25, granddaughter).

Participants rarely mentioned the term “palliative,” even if they were assisted in the PCU, due to a pervasive lack of awareness regarding the concept of terminality (the opacity we interpreted). This prevented any discussions surrounding palliative interventions.

However, as health deteriorated and active treatments ceased, some patients began envisioning a return home, particularly if they perceived a loss of purpose in remaining in Italy. In these cases, an alternative strategy emerged: the transnational circulation of care. Some patients sought to bring medications back to their country of origin or planned to return periodically to Italy for follow-ups in oncological services.

“The doctor gave me extra medication for the trip in case I could not return soon” (P21, patient).

This approach was often driven by PC specialists’ understanding of the lack of essential medications in their home countries or the high cost of medical treatments, which interviewees frequently complained were inaccessible without significant financial resources. For these patients, sustaining a connection with the Italian healthcare system constituted a fundamental strategy for securing uninterrupted access to oncological care.

Thus, healthcare was a dynamic rather than static force. It initially served as a strong anchor for remaining in Italy, but gradually faded in influence as curative treatments ended and alternative strategies, such as transnational care, emerged. In this process, the availability and accessibility of pain management played a crucial role, as patients evaluated whether adequate treatments were available in their home country and whether they felt safe and reassured that their pain would be effectively controlled.

Finally, the feasibility of transporting medications across borders was a key factor, as patients sought reassurance that they could continue their treatment regimen without interruptions, ensuring continuity of care and maintaining a sense of security in their symptom management, regardless of location.

### Bureaucratic and financial barriers as an obstructing force: resistance in the gradient

Bureaucratic complexities significantly shaped the decision-making process, often as barriers impeding timely and autonomous EoL care and burial choices. Patients and families frequently faced challenges related to disability benefits, residence permits, healthcare access, and the repatriation of remains.

“I went to do the ‘730’ document [*name of the form to fill out for paying taxes*], but they asked me for the doctor’s certificate, so I had to postpone it. I didn’t even know that was needed, and if the hospital case manager hadn’t helped, I would have been lost” (P01, widow).

Legal status and material conditions also influenced the ability to have family members close or to visit relatives abroad, directly affecting EoL preferences.

The complex entanglement between legal status and bureaucratic procedures emerged concerning access to social welfare. Participant P21 offered a striking example: although he had officially been granted a disability-related financial benefit—an economic support measure provided by the Italian welfare system for individuals with certified medical conditions—he was nonetheless unable to receive it due to the lack of a valid legal residence.

“Now that I need to come back here to Italy [*from Ghana*] for health reasons… I have nothing here. I don’t have a residence. I was granted disability, but I still have nothing in my hands. Without at least a legal residence, it’s a problem” (P21, patient).

The bureaucratic burden often weighed most on patients and families at their most vulnerable moments, exacerbating stress and uncertainty. This was particularly challenging due to the lack of a clear, centralized pathway for obtaining essential information. Many felt lost and uncertain about where to seek guidance, forced to navigate multiple offices and institutions to piece together the necessary documentation and resources. The complexity of these administrative processes delayed decision-making and intensified the emotional and logistical strain on families already coping with the demands of EoL care.

Economic constraints exacerbated these difficulties, especially when patients wished to return home but lacked financial resources. The cost of travel and repatriation was often prohibitive.

“She was comfortable in hospice, but leaving was difficult. The financial cost of keeping her here and bringing her daughters from Ukraine was substantial. Yet, her husband valued her well-being more than anything. He feared she wouldn’t receive the same care back home” (P10, cultural mediator).

“Initially, he believed there was still hope. Despite his wife’s insistence on returning to China, they lacked the funds. They had to purchase the tickets, and upon arriving in China, it was necessary to enter the hospital” (P15, cultural mediator).

However, community solidarity sometimes provided relief. Some participants who could afford it reported paying for insurance after settling in the host country.

“Everyone, in my opinion, would have returned home willingly… but more than anything else, the economic problems, the travel, the expenses…” (P12, charity director).

“Many move ahead because they take out insurance covering the funeral service and repatriation. Let’s say that those who consider it get ahead of the game. Also, from personal experience, my parents have been doing this for years… Actually, I speak for the Muslim community. When a family has no financial means, the entire community steps in. It’s not just the family that bears the expenses; in most funerals, it’s the whole community that provides support, so family members are not left without assistance, burdened” (P25, granddaughter).

Funeral services also played a crucial role, supporting families in repatriating the body by handling complex bureaucratic procedures. Additionally, they often ensured respect for cultural traditions, such as preparing the body for transport using specific preservation techniques.

“Yes, in fact, when the bodies are transported long distances, we also perform preservative injections, specifically formalin, to ensure their preservation, as in many cases they are reopened upon arrival” (P26, funeral operator).

Additionally, cultural mediators perceived themselves as crucial in assisting and supporting navigating bureaucratic obstacles.

“So we spent quite a bit of time figuring out how to bring it back home to China and reaching out to the Chinese associations. They do a lot of great work, but in the end, it’s always the consulate that can really help. So, we got in touch with them, and I asked for guidance on filling out the forms online and all the information involved.” (P15, cultural mediator).

Bureaucratic and financial challenges, though not directly related to the cost of PC, functioned as significant obstructive forces, often delaying or limiting EoL options. These obstacles included legal residency, repatriation documentation, and social benefits access. In many cases, cultural mediators, charitable organizations, and funeral service providers played a crucial role in alleviating these burdens, helping families navigate complex systems during an already vulnerable time.

### Social networks as a stabilizing force

Social networks functioned as a balancing force within the decisional gradient field, stabilizing decisions depending on where familial support was most potent. The presence of family members—both in Italy and/or in the country of origin—was critical in shaping the patient’s EoL preferences.

“My family visits me (referring to children in Italy)… and those who are far away (two and a half hours by plane from Morocco) support and encourage me” (P18, patient).

When treatment ended, families frequently had an essential role in deciding whether the patient would stay or return home.

Decisions about funeral and burial locations were also often profoundly influenced by the family:

“Yes, it’s the families who make the choices, not that someone imposes them… they are family choices… (There are) those who wait for family members from afar to show (the body), and without them, they don’t proceed to make the funeral” (P27, funeral operator).

Spiritual and religious beliefs emerged as significant elements in shaping preferences around burial and the meaning of place at EoL. For some participants, faith traditions provided existential reassurance and continuity across geographic boundaries. As one cultural mediator recounted, faith could mitigate the distress of physical dislocation at EoL, offering a unifying worldview that softened the tension between homeland and host country.

“He [the patient’s husband] told me about his daughters, saying that he felt sorrow because he was here while they were there, and that he would have liked to return home. But then he added, ‘It does not matter in the end; the earth belongs to God, and whether I am here or there, it is the same.’” (P10, cultural mediator about a Catholic Ukrainian husband of a patient).

At the same time, preferences around burial also reflected deeply embedded cultural and religious practices tied to identity, community, and ritual proximity. A participant of Muslim background (P25) reflected on how the availability of designated Muslim burial spaces in Italy had shifted some families’ choices over time.

“Because in any case, knowing that you are buried in Muslim territory among Muslims has a different value. It is true that even in Italy, great steps have been taken. For example, in Reggio Emilia, we have a Muslim camp that I think many years ago did not exist. In my opinion, it is also this that has pushed many Muslims to choose, instead of repatriation, to be buried here too. It’s a mix; many things are going on. But obviously for Muslims, religion… But others choose to stay here for the love and connections. For instance, my aunt says, ‘If I die, I want to be buried here because I know my children live here.’ (P25, granddaughter).

This preference was shaped by two interrelated aspects: the desire to rest in the same land as their ancestors and deceased relatives, and the need to be buried where surviving family members could regularly visit and perform the necessary rituals of remembrance and/or devotion. Thus, burial location was a matter of personal or ancestral/spiritual connection and ensuring accessibility for loved ones to uphold spiritual and cultural traditions.

“The decision regarding burial in Morocco? She would have made it herself. She was born there and spent her childhood in Morocco. She moved to Italy at the age of 28. She wished to be buried close to her mother, by tradition, as she came from a close-knit and strongly connected family. She was also a religious person.” (P03, daughter).

The desire for repatriation after death emerged as a strong theme across several narratives, reflecting the enduring cultural and symbolic importance of the homeland for many migrant families. Decisions about burial location were often guided not only by religious or traditional values, but also by relational ties and a sense of continuity with one’s place of origin. This practice transcended the length of time spent in the host country or even generational status, as highlighted by one funeral operator, who noted:

“I could say that, although I don’t have precise numbers, migrants tend to return traditionally. For example, 99% return to Nigeria, whether they’ve been in Italy for one, two, three, five, ten, twenty, or thirty years. Eastern countries like Moldova and the former Soviet Republics see almost all return. Whether they come back as corpses or ashes doesn’t change much; the important thing is that they return. Very few remain, even if they are second or even third generation. It’s likely their custom, but I’m not sure if it’s really a ritual or if they still have family members there” (P28, funeral operator).

Lastly, several participants voiced their worries about posthumous repatriation of their loved ones, expressing reluctance to place a financial strain on families, since the costs of repatriating a body after death are considerably greater than the expenses associated with travel when someone is alive.

### Settling the gradient’s forces

The core category, “settling the gradient’s forces,” signifies the culmination of the decision-making process. This notion emerged as a shared concern among all participants involved in harmonizing the divergent forces represented by this decisional gradient. This gradient denotes the point at which the competing influences affecting the circumstances of the dying individual reach a state of balance. In many instances, death precluded the possibility of finding a resolution. However, when action was still feasible, outcomes were more likely to be achieved if bureaucratic and financial constraints were overcome, social networks across the country of residence and origin were activated, and healthcare demands were successfully negotiated within these interconnected social and institutional frameworks.

Additionally, in situations where patients were unaware of their terminality, decision-making was often transferred to HPs and families. This shift expanded the decision-making network, with medical teams working alongside families to determine the most appropriate action.

“In some cases, the patient does not know, and then the decision is in the hands of the family and the doctors. It is a delicate balance, where we have to navigate both medical needs and the family’s wishes” (P13, association member).

## Discussion

This study highlights the intricate interplay of structural, cultural, and social factors influencing the decision-making process regarding the place of care and death for MCPs in Italy. Numerous studies have highlighted the crucial role of these factors in EoL decision-making, yet a comprehensive examination of how they interact has remained largely unexplored [25]. This interaction unfolded in our study as “settling the gradient” of the factors (forces) at play: it was the primary concern of all participants to achieve a reconciliation among healthcare needs, bureaucratic and financial constraints, shared cultural beliefs, and preferences, all often within the context of patients’ unawareness of terminality and relative illiteracy regarding the nature and the role of PC.

Responding to healthcare needs emerged as a crucial stabilizing force, sometimes even an attracting one, where the availability of quality care (predominantly curative) in the host country may have encouraged patients to remain. Conversely, bureaucratic and financial constraints acted as obstructing forces, complicating access to care, social benefits, and repatriation. Social networks played a pivotal role in shaping these dynamics, influencing whether the support structures in the host or country of origin had more significant sway over final decisions. Within this last category, we understood that networks also worked for spirituality and cultural values, often reinforcing the preference for family-based decision-making over individual autonomy. Ultimately, these forces operated within a dynamic continuum, where the final resolution—whether to remain in the host country, return to the country of origin, or move freely between the two—was determined by the relative strength of pulling, stabilizing, and resisting forces.

Our participants appreciated the overall healthcare system in Italy, noting the timely access to oncological care and the availability of treatments, often compared to the situation in their home countries, as highlighted elsewhere [56, 57]. Many migrants may view the care received in the host nation as superior to that in their countries of origin [31, 57, 58], becoming a factor influencing decision-making, attracting foreign patients, and contributing to what is described as “health tourism” [22]. However, communication challenges, cultural barriers [22, 59], and difficulties in engaging with services and facilities [19, 60–62] persist. These challenges stem from language barriers, cultural mediators/interpreters shortages [22], lack of professional training in culturally competent care, and miscommunication.

Although the PCU staff had contacted all patients, PC was never mentioned by the patients during interviews and was only sporadically discussed by other participants. This is consistent with earlier studies indicating that migrant patients may have a limited grasp [13, 36, 63, 64] or taboos regarding PC [65, 66]. In the MCPs’ PC context, specialists should recognize that migration history and PC awareness significantly influence participants’ ability to engage with EoL care [67]. Different studies highlighted in this regard that low PC literacy impedes effective communication between MCPs and PC specialists, does not foster patient agency [68, 69] and learning [70, 71], and delays critical decisions.

Additionally, our findings revealed a significant degree of patient unawareness or explicit acceptance regarding their terminal diagnosis, a phenomenon we have described as the “opacity of terminality.” In our study, this opacity included a systemic and relational silence around terminality. Cultural norms of protection and language barriers contributed to a communication environment where full disclosure was often withheld or delayed. Consequently, decision-making about the place of care and death was frequently led by families or proxies, sometimes in consultation with HPs, rather than by patients themselves. This dynamic can be seen as a form of collective decision-making [72, 73], where families act as moral agents on behalf of the patient [74], especially when death is considered too heavy a burden to disclose [22]. While this challenges Western focus on individual autonomy [75], often embedded in the bio-medical ethos, it also highlights the importance of understanding how relational autonomy and culturally mediated communication shape EoL choices. The forces identified in our gradient model still influenced patients’ experiences, often through their families and caregivers.

The literature has extensively documented the communication challenges HPs face, stemming from language barriers and a lack of cultural sensitivity [13, 58, 60, 76].

Building meaningful relationships with patients and their families is essential before initiating open discussions about terminality [71, 77, 78], which the patients we interviewed did not express awareness of. HPs should adopt a cultural humility [79, 80] stance to understand cultural differences and adapt communication strategies. This ensures that conversations about prognosis and EoL care align with patients’ values, beliefs, and readiness to engage with such topics [81]. In this context, the role of cultural mediators is essential [52, 82], and HPs must receive training in effective collaboration with these professionals. Their involvement has become necessary to ensure respectful and equitable care, facilitating a highly individualized approach for MCPs [13, 22, 83].

Furthermore, integrating early training regarding collaboration with cultural mediators may significantly improve communication practices and outcomes during EoL care [84, 85].

This study has taught us that establishing and continually improving interactions between PC specialists and migrant communities cannot be delayed. Proactive outreach and constructive dialogue can strengthen trust, fostering mutual understanding and collaboration [73]. Furthermore, promoting a well-aware acceptance of a PC approach may serve as an effective strategy to unlock PC’s full potential. It can help reduce suffering, enhance comprehension and acceptability of prognosis, and support MCPs and their families in navigating choices. All this is most effective when patients are aware and informed [29, 86, 87].

This could open the possibility of implementing advance care planning (ACP) conversations, where the place of death should be an integral aspect for MCPs to address, enabling them to express their wishes and avoid unwanted transitions. Yet, these conversations remain underdeveloped in PC practice, particularly in multicultural settings [88]. It has been observed [58, 64, 81] that professionals regard the MCP’s approach to death as an event that takes form independently of an individual’s culture of origin and specific life path, including migration. By adopting this perspective, they detach the concept of death from cultural, geographical, and biographical contexts. The research conducted by Green and colleagues indicates that the preferred place of death was documented in merely 45% of patient records [18]. Furthermore, only 20% of these records included a report of discussions with nursing staff regarding the care of the deceased’s body [81]. While discussing topics like death and burial can be challenging, there is no doubt that these conversations are highly relevant to PC and could find a fitting place in ACP by incorporating geographical, cultural, and social considerations mediated by migration into their care frameworks.

Our findings indicate that ACP conversations with MCPs should account for the specific characteristics of our study populations, particularly the influential “force” of networks. In our study, decision-making was a collaborative effort between medical teams and family members, highlighting the importance of collective input [23, 89–93], especially when patients were unaware of their terminal conditions. This communal approach to decision-making contrasts with the Western paradigms of autonomy and self-determination [58, 61, 94], which often emphasize individual choice separate from relational contexts [36]. Anthropological studies have suggested the ideological nature of this paradigm even in Western societies, and the profoundly relational nature of identities and choices, especially about deeply unsettling experiences of illness/health and EoL care [75, 95, 96]. The centrality of relations in decision-making takes even more complex facets in migrant patients and their networks, influenced by identity, cultural, systemic, bureaucratic, and financial factors, shaped by migration [58]. Thus, we recommend a thorough reevaluation of autonomy in PC. PC specialists must actively adjust PC principles to fit the patient’s relational context, recognizing that decision-making may occur collaboratively instead of solely individually, even in native contexts. Concerning migrant patients, this requires reinterpreting autonomy-related frameworks to reflect individual and cultural values, family structures and dynamics, and the role of transnational support networks, ensuring that PC approaches remain patient-centered and culturally responsive [73]. Furthermore, the unique experiences of migration should be integrated with culturally appropriate decision-making approaches to ensure equitable access to ACP for migrant groups [97, 98].

By examining how the MCPs’ networks operate within the decision-making process regarding the place of dying and death, we also gained insight into how a newer approach, such as transnationalism, could enhance PC provision to MCPs. Transnationalism describes how individuals maintain social, economic, and cultural ties to their countries of origin while living elsewhere [99–101].

Leveraging on this approach, a distinction can be made between the place of dying and the place of death, with important implications for how PC is conceptualized and delivered. While the place of death is often understood as a definitive location where life ends—typically documented as a hospital, home, or hospice—the place of dying may be more appropriately viewed as a process unfolding across time and space. Many participants described patterns of transnational movement, or the desire for such mobility, in which care, relational ties, and emotional attachments extended across borders. In this sense, dying was not experienced as a fixed moment in a single place, but rather a fluid and negotiated process shaped by shifting clinical [41], bureaucratic, and social conditions. This fluidity challenges the assumption of a linear, place-bound trajectory of dying. It underscores the need for palliative care models to accommodate temporal and spatial complexities, particularly for migrant populations. Recognizing dying as a transnational and processual experience invites further reflection on how to support patients and families in navigating multiple “places” of care, identity, and belonging in the final stages of life.

Our findings underscore the integral role of family and community networks, often transnational, in shaping EoL decisions and supporting patients’ emotional well-being within diasporic contexts. While the length of time migrants spend in a host country might influence their care and death preferences, this factor was not a significant theme in participants’ narratives. Instead, the quality of relationships—like family ties, employment stability, and social integration—played a more substantial role. Although these connections develop over time, they don’t always align with the duration of stay. For example, participants who moved to Italy for cancer treatment had short stays yet preferred to remain due to perceived care quality.

In contrast, some long-term residents wished to return to their home countries for cultural or familial reasons. These findings indicate that while time since migration interacts with social networks and bureaucratic rights, it is not a direct predictor of preferences for EoL care. Future research should examine how the duration of stay relates to these relational and structural factors across migrant groups.

Adopting a transnational approach within PC may foster the integration of diverse practices and values, particularly relevant for MCPs who may experience an imaginative and corporeal legacy of transnational belonging [102]. The role and involvement of family in their country of origin is a central theme of our study, consistent with recent research findings. A scoping review by Gerber and colleagues [36] highlights that migrant patients in the late stages of life often experience feelings of being caught between two contexts. These individuals conveyed challenges in reconciling the expectations of their previous and current homes, a phenomenon referred to as the concept of a “double home experience” [35], confirming the need for a transnational approach for PC.

The transnational approach we highlighted identifies systemic obstacles, including bureaucratic and financial barriers, which often delay or limit options at the EoL. These challenges significantly hinder fulfilling EoL needs [14, 36]. Periyakoil and colleagues [60] explained how a lack of finances and insurance can affect MCPs’ care at EoL. In our study, we clarified how these problems may affect the possibility of repatriation or bringing family members closer together for the EoL. However, these barriers can be alleviated through interventions by transnational networks, such as initiatives to collect funds for repatriation [22]. Our findings emphasize that as patients’ health deteriorated and active treatments were discontinued, many individuals began to envision a return to their home country, mainly when they felt a diminished sense of purpose in remaining in Italy. While our findings suggest that burial in the country of origin is a significant preference among many MCPs, our sample was not statistically representative, and we did not explore differences related to other diagnoses. Nevertheless, this phenomenon merits further investigation in a region such as Emilia-Romagna, where the incidence of foreign residents is particularly high. Future research could explore the prevalence, logistics, and emotional dimensions of post-mortem repatriation and how this intersects with healthcare planning and EoL support for diverse migrant populations.

### A proposal: a tool to explore the desires and possibilities of migrant patients, facilitating decision-making regarding EoL care

To effectively care for MCPs, PC teams must be equipped to bridge the gap between medical, social, and bureaucratic challenges. Based on our findings, this requires moving beyond the traditional clinical team composition. PC teams caring for MCPs should include professionals with specific training in intercultural communication and be supported by cultural mediators and, where possible, ethnopsychologists [103, 104] or medical anthropologists as consultants [105]. These figures are crucial in facilitating culturally sensitive dialogue and building the capacity of professionals along the way, navigating transnational family dynamics, and interpreting complex emotional and spiritual needs shaped by migration experiences. With such expertise integrated into the team, PC can serve as a mediating force within the decisional “gradient,” helping patients and families reconcile the multiple structural, relational, and symbolic forces at play, and ultimately supporting compassionate, person- and family-centered care regardless of the final place of death.

To assist PC specialists in addressing the challenges encountered when caring for MCPs at the EoL, we drew inspiration from Deamant and colleagues’ Checklist to Facilitate International Travel for Patients at the End of Life [14]. Their checklist, developed from best practices, resources for HPs, and recommendations, offers practical guidance for HPs supporting terminally ill cancer patients navigating international travel. It includes symptom management, documentation, logistical planning, and transnational care coordination considerations. In a similar spirit, and grounded in the findings of our study, we developed a preliminary question guide to support HPs during PC consultations with MCPs. The authors developed the proposed questions collaboratively through an iterative discussion and reflection on the study’s main findings. Drawing from the most salient categories, the team translated these insights into exploratory prompts to guide culturally sensitive conversations in palliative care consultations.

Unlike Deamant et al.‘s structured checklist, our proposal should be considered a tentative and exploratory tool—a starting point to facilitate culturally sensitive conversations about care preferences, logistical constraints, and the emotional and symbolic meanings associated with place, family, and migration. This guide (Table 3) has not yet been validated. Still, it represents an effort to operationalize our qualitative insights and the clinical expertise of the authors into a format that may assist clinicians in practice. It highlights domains that emerged as particularly significant in our participants’ narratives and invites further development and testing through collaborative research and clinical application.

**Table 3:** Guiding questions for PC professionals assisting CMPs at eol

Topic	Proposed questions
**Approaching Palliative**	- What did you or your family understood of the type of care (palliative care) that you will be receiving from now on? - What did you or your family understood about receiving oncological medications/therapies? - What is most important to you/your family at this moment? - Where would you prefer to continue your care? - What are the key issues you would like to discuss with us now? - How can we support you in making decisions about your care, including where you would like to receive it and, if you have already thought about it, where you would prefer to be in the next weeks?
**Care needs**	- How do you feel that your symptoms are currently well controlled? - How adequately managed do you believe your symptoms would be in your country of origin? - To what degree is medical treatment and necessary medication available in your home country and accessible or free of charge? - How safe would you feel safe if we prescribed and provided you with the necessary medications for your care? - How can we better assist you in managing your symptoms and treatment?
**Social resources**	- How do you feel supported by your family or loved ones? - Would you like us to speak with your family members here or in your home country via phone or video call? - What would you like us to discuss with them? - Would you prefer to have a cultural mediator present during these conversations? - If you decide to continue care in your country of origin, do you have a loved one who could accompany you on the trip?
**Financial and Bureaucratic Considerations**	- How do you feel about the necessary financial resources to travel if you choose to return home/to stay here if you choose to stay? - Are you facing any difficulties with documents, visas, or passports? Which ones? - Who could assist you in these aspects (e.g. a family member, case manager, or the foreign office)? To what degree have you already discussed this with them? - Would you need a medical certificate translated into your language? - How can we help you navigate any financial or bureaucratic challenges?
**Emotional dimension**	- Are there times when you feel improved? If yes, would you like to share those experiences with us? - Who or what currently provides you the most support (e.g., family, spiritual guidance…)? In what way? - Do you have any specific thoughts or concerns you would like to discuss with us?
**Other** **issues**	-Would you like to stay in contact with us by e-mail or telephone in the next period if you decide go home? - Would you like us to contact the healthcare professionals you will be seeing at home? - Have you any other doubt/question for us?

Additional research is required to enhance and validate this guide and clarify its application, whether with patients and/or family members. Future studies could pilot-test these questions with key informants, including MCPs, families, and HPs, to assess their comprehensibility, acceptability, and effectiveness in clinical practice. Additionally, it would be valuable to develop a complementary prompt list from the perspective of patients and their networks, providing them with structured ways to express their concerns and preferences during consultations. For example, prompts could include articulating personal goals, cultural needs, and practical challenges when communicating with HPs.

### Strengths and limitations

This study provides an in-depth exploration of the decision-making processes of MCPs at the EoL. It uses a qualitative approach that allows for the capture of rich, context-specific narratives. A key strength of this methodology is its ability to highlight the interplay of cultural, social, and systemic factors, offering insights that may not emerge through quantitative methods. GT enabled an iterative and inductive analysis, ensuring that the emergent themes were deeply rooted in participants’ experiences.

However, several limitations must be acknowledged. The study sample was limited to a specific geographic and healthcare context, which may restrict the findings’ applicability to other migrant populations or healthcare systems.

Notably, only seven patients were enrolled in the study, representing 12% of the total cases managed by PCU in 2024. This highlights not just the limited sample size but also the critical role these cases play in understanding the broader implications of our research. Thirdly, while efforts were made to include diverse voices, language barriers and differences in health literacy may have influenced the depth of engagement with some participants. Then, the reliance on proxies for decision-making insights, particularly in cases where patients were unaware of their terminality, may also have introduced interpretative bias.

From a methodological perspective, the interview guide was neither pilot-tested nor reviewed by external experts or patient representatives before use, which we acknowledge as a methodological limitation. While cultural mediation was essential for ensuring inclusive participation and facilitating trust in three cases, it also introduced an additional interpretive layer in the data collection process while conducting interviews. Mediators may have shaped how questions were received and how responses were expressed. Although the research team employed reflexive strategies to account for these dynamics, we acknowledge that the presence and translation by mediators influenced the data’s depth, nuance, and length of the three interviews.

## Conclusions

This study highlights the complex and multi-layered challenges shaping EoL decision-making for MCPs and their place of death. It demonstrates how healthcare systems, social networks, bureaucratic constraints, and cultural beliefs intersect profoundly. Addressing the unique needs of MCPs requires an integrated approach that amalgamates clinical, social, and transnational dimensions of care. Recognizing EoL decision-making as a gradient, where evolving constraints and opportunities shape choices, can help HPs and policymakers develop more responsive interventions. Healthcare institutions can empower patients and families to navigate this complex EoL landscape with greater dignity and agency by attempting to reduce bureaucratic barriers and strengthening cross-border support systems. At the same time, PC specialists must embrace more inclusive and culturally responsive care models that respect diverse factors influencing decision-making, caregiving, and EoL preferences. Thoughtfully designed PC interventions can shift the balance of this decision-making gradient by reinforcing the feasibility of remaining in the host country or mitigating repatriation’s emotional and logistical challenges.

To achieve these goals, PC services should prioritize culturally sensitive communication and enhance transnational family/community engagement. Future research should further explore how to integrate culturally tailored decision-making frameworks into PC practices, ensuring that all patients, regardless of migration status, receive dignified, equitable EoL care.

## Electronic supplementary material

Below is the link to the electronic supplementary material.

Supplementary Material 1

Supplementary Material 2

## Data Availability

The data supporting this study’s findings are not openly available due to sensitivity reasons but are available from the corresponding author upon reasonable request. They are located in controlled access storage at the Qualitative Research Unit, Azienda USL-IRCCS of Reggio Emilia (Italy).

## Abbreviations

ACP: Advance Care Planning
EoL: End of Life
EU: European Union
GT: Grounded Theory
HP: Healthcare Professional
MCP: Migrant Cancer Patient
PC: Palliative Care
PCU: Palliative Care Unit
QoL: Quality of Life
SRQR: Standards for Reporting Qualitative Research
