# Supplementary figures and images for: Between host country and homeland: a grounded theory study on place of dying and death in migrant cancer patients
Source: BMC Palliat Care. 2025 Aug 8;24:220. doi: 10.1186/s12904-025-01821-3 (PMC12335150; doi:10.1186/s12904-025-01821-3)

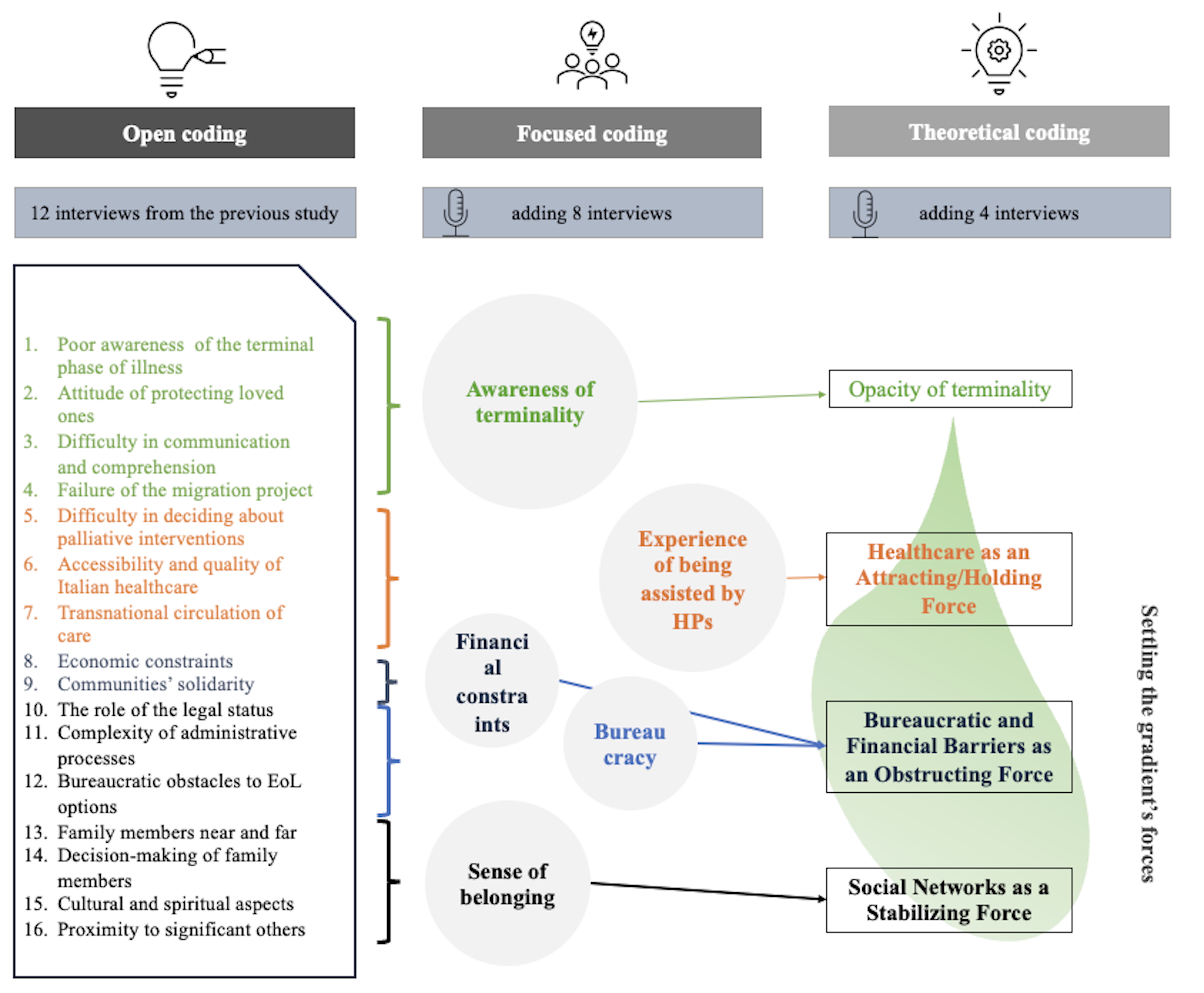

Supplement: Supplementary file 2 — Supplementary Material 2 [file 12904_2025_1821_MOESM2_ESM.jpg]
